# Determining an optimal cut-off value for follicle-stimulating hormone to predict microsurgical testicular sperm extraction outcome in patients with non-obstructive azoospermia

**DOI:** 10.20945/2359-3997000000217

**Published:** 2020-03-18

**Authors:** Bahia Namavar Jahromi, Shahryar Zeyghami, Mohammad Ebrahim Parsanezhad, Parvin Ghaemmaghami, Afsoon Zarei, Maryam Azizi kutenaee, Parastoo Sohail, Pedram Keshavarz

**Affiliations:** 1 Infertility Research Center Department of OB-GYN Shiraz University of Medical Sciences Shiraz Iran Infertility Research Center, Department of OB-GYN, Shiraz University of Medical Sciences, Shiraz, Iran; 2 Infertility Research Center Shiraz University of Medical Sciences Ghadir madar Hospital Shiraz Iran Infertility Research Center, Shiraz University of Medical Sciences, Ghadir madar Hospital, Quran Blvd., Shiraz, Iran; 3 Hormozgan Fertility and Infertility Research Center Shiraz University of Medical Sciences Ghadir madar Hospital Shiraz Iran Hormozgan Fertility and Infertility Research Center, Shiraz University of Medical Sciences, Ghadir madar Hospital, Qorand Blvd., Shiraz, Iran; 4 Departments of biostatistics medical school Shiraz University of Medical Sciences Shiraz Iran Departments of biostatistics, medical school Shiraz University of Medical Sciences, Shiraz, Iran; 5 Department of Radiology Medical Imaging Research Center Shiraz University of Medical Sciences Shiraz Iran Department of Radiology, Medical Imaging Research Center, Shiraz University of Medical Sciences, Shiraz, Iran

**Keywords:** Follicle-stimulating hormone (FSH, non-obstructive azoospermia (NOA, microsurgical testicular sperm extraction (micro-TESE

## Abstract

**Objective:**

To determine the optimal cut-off value for follicle stimulating hormone (FSH) to predict the outcome of microsurgical testicular sperm extraction (micro-TESE) in patients with nonobstructive azoospermia (NOA).

**Subjects and methods:**

We included a total number of 180 patients with NOA. The serum level of FSH was determined and all the subjects underwent micro-TESE. We determined the optimal cut-off value for FSH and assessed whether the test could be effectively used as a successful predictor of sperm retrieval by calculating the Receiver Operating Characteristic (ROC) area under the curve.

**Results:**

Overall we included a total number of 171 patients with mean age of 34.3 ± 8.6 years. The micro-TESE was considered to be successful in 79 (43.8%) while it failed in 92 (56.2%) patients. We found that the mean level of serum FSH was significantly higher in group those with failed micro-TEST compared to successful group (p < 0.001). The cut-off value for FSH was calculated to be 14.6 mIU/mL to predictive the outcome of micro-TESE with a sensitivity of 83.5% [73.5%-90.9%] and a specificity of 80.3% [69.5%-88.5%]. At this value, the other parameters were calculated to be PPV, 81.5%; NPV, 82.4; LR+, 4.23; and LR-, 0.21.

**Conclusions:**

The results of the current study indicate that FSH plasma levels above 14.6 mIU/mL can be considered to be the failure predictor of the micro-TESE in NOA patients.

## INTRODUCTION

Evaluation of testicular function is an important and compulsory aspect of management of infertility (1). The initiation and maintenance of spermatogenesis is regulated through the hypothalamic-pituitary-gonadal axis and is mainly dependent on the follicle-stimulating hormone (FSH) and androgen (1-3). Production of FSH and Luteinizing Hormone (LH) from the anterior lobe of the pituitary gland is stimulated by the Gonadotropin-Releasing Hormone (GnRH) secreted from the hypothalamus (1). FSH affects the Sertoli cells of the seminiferous tubules to continue normal spermatogenesis. Production of the inhibin B from the spermatogenic cells suppresses FSH secretion from the pituitary gland (4). Plasma FSH levels usually have an inverse relationship with spermatogenesis; production of inhibin B is reduced and plasma FSH level is increased in spermatogenic failure disorder. Scientific reports have revealed 10-15% prevalence of infertility issues in the general population (5), with almost 50% of the cases being relevant to a male factor (6,7). Azoospermia, with the prevalence of 10-20% in infertile male population can be clinically classified as obstructive (post-testicular) and non-obstructive (pretesticular or testicular) (6). Non-obstructive azoospermia (NOA) is more common compared to obstructive azoospermia (OA) and occurs in 80-85% of men with azoospermia (6). Although NOA has been described as damaged sperm production of the whole testis, focal normal spermatogenesis can be observed in 50-60% of men with NOA (8).

Currently, use of assisted reproductive techniques (ARTs) is an acceptable approach for men with azoospermia and testicular atrophy. Using endocrine markers to predict the success of sperm retrieval for infertile men before using ARTs can decrease the costs. In this respect, elevated FSH levels, as an inexpensive and non-invasive method, is considered to be a clinically suitable marker in assessment of infertile men. Unfortunately, there is no consensus regarding an optimal cut-off value for FSH to predict the existence of spermatogenesis in patients with NOA. Only few studies have addressed the cut-off values for FSH in patients with NOA undergoing sperm retrieval (9,10). In addition, some studies have demonstrated that FSH is not an indicator of successful sperm retrieval in these patients (11,12). But controversy still exists regarding the value of FSH in predicting the outcome of sperm retrieval in patients with NOA. Thus, the aim of the current study was to determine the optimal cut-off value for FSH to predict the outcome of microsurgical testicular sperm extraction (micro-TESE) in patients with NOA.

## SUBJECTS AND METHODS

### Study population

This prospective cross-sectional study was carried in our center during a 7-month period from April 2015 to October 2015. We included a total number of 171 consecutive subfertile men of Iranian nationality referring to the Infertility and Embryology center of Shiraz University of Medical Sciences. All the patients were between 30 to 45 years of age and were diagnosed to suffer from NOA. The inclusion criteria were infertility defined as no achievement of a pregnancy after 12 months of unprotected intercourse, two spermiograms showing azoospermia with time interval of at least 70 days. We excluded the patients with fever, those using hormonal preparations during the last 3 months, those who had undergone vasectomy, and those with vas deference agenesis determined by physical examinations. We also excluded those with hypogonadotropic hypogonadism. The study protocol was approved by either the institutional review board (IRB) and the medical ethics committee of Shiraz University of Medical Sciences. All the patients provided their informed written consents before inclusion in the study.

### Study protocol

All the patients underwent complete andrologic evaluation, including history, physical examination, hormonal profile (serum FSH, LH, total Testosterone, prolactin), and spermiogram. All subfertile men underwent testicular FNA. Imaging (scrotum ultrasonography, color Doppler, pelvic computed tomography) and genetic (karyotype, Yq microdeletions) studies were performed in subfertile men as clinically indicated.

Sperm was obtained by masturbation after 3 to 5 days of abstinence. The samples were centrifuged at 600 × g for 10 minutes, and if no sperm were detected in the pellet a diagnosis of azoospermia was confirmed. Sperm concentration, motility, and morphology were evaluated according to the World Health Organization criteria (13). Blood samples were obtained at 9 AM and centrifuged for 20 minutes. The serum was separated and stored at –20°C until analysis was performed. The serum level of FSH was measured by chemiluminescent immunometric assay using commercial kits (IMMULITE 2000 FSH, Deerfield, IL USA).

### Micro-TESE procedure

Standard micro-TESE surgery was operated by an expert surgeon under operative microscope to avoid testicular vessel destruction according to the Schlegel technique (14). Sperm processing was performed meticulously in an operating room via mechanical dissection of seminiferous tubules using insulin needles in order to increase sperm retrieval rate during the intra-operative period (15). All the samples were stained using hematoxylin and eosin (H&E) according to the standard protocol (16). By examining two separate microscopic slides, having at least 100 different tubule sections, the Meng classification system (17), based on the predominant type of cells was used by an expert pathologist who was blind regarding clinical information of the patients.

1. Normal spermatogenesis: Cells from all the stages of spermatogenesis were detected in adequate number.
2. Hypospermatogenesis: Although cells from all the stages of spermatogenesis (including sperm) were encountered, their number was significantly reduced.
3. Maturation arrest: Sperm maturation stopped in early stages of spermatogenesis. Neither sperm nor spermatids were detected (‘‘complete maturation arrest’’). In case occasional sperm were found in any of the four FNA sites, the case was classified as ‘‘incomplete maturation arrest’’.
4. Sertoli cell–only syndrome (SCOS): Spermatogenesis cells were completely absent, Sertoli cells being the only cells detected (‘‘complete SCOS’’). In case occasional sperm were found in any of the four FNA sites, the case was classified as ‘‘incomplete SCOS’’.

Thus, in this classification system, six possible FNA diagnoses exist; in four (normal spermatogenesis, hypospermatogenesis, incomplete maturation arrest, and incomplete SCOS) sperm are present, whereas in the remaining two (complete maturation arrest and complete SCOS) sperm are absent. Successful sperm retrieval was considered the aforementioned four categories (group A) and the failed micro-TESE was considered to be the last two categories (group B).

### Statistical analysis

All results are presented as mean ± Standard Deviation (SD) and proportions as appropriate. The Student t test was used to compare serum FSH levels between groups A and B. P values less than 0.05 were considered significant. These statistical analysis was performed using statistical package for social sciences (SPSS Inc., Chicago, Illinois, USA), version 18.0. Receiver Operating Characteristic (ROC) curve was used to determine the optimal-off value for FSH to assess whether the test could adequately discriminate between groups A and B. The ability of serum FSH to predict sperm retrieval was estimated based on sensitivity, specificity and area under the ROC curve (AUC) at various cut-off values. An AUC of 1 indicates perfect discrimination, AUC > 0.9 shows high accuracy and AUC between 0.7 and 0.9 indicates moderate accuracy, whereas an area of 0.5 indicates that the test discriminates no better than chance (18). In addition, positive and negative predictive values (PPV, NPV), and positive and negative likelihood ratios (LR+, LR-) were calculated. LR+ was defined as [sensitivity/ (1-specificity)], while LR- was calculated as [specificity/ (1-sensitivity)]. These analyses were performed using STATA 12 software (StataCorp LP).

## RESULTS

Overall we included a total number of 171 patients with mean age of 34.3 ± 8.6 years. The baseline characteristics of the patients are summarized in Table 1. There was no significant difference between two study groups regarding the baseline characteristics. As demonstrated, the micro-TESE was considered to be successful in 79 (43.8%) while it failed in 92 (56.2%) patients. We found that the mean level of serum FSH was significantly higher in group those with failed micro-TEST compared to successful group (p < 0.001). other parameters were comparable between two study groups (Table 2).

**Table 1:** The baseline characteristics of 171 subfertile men with non-obstructive azoospermia included in the current study

Variable	Value
Age (years)	34.32 ± 8.61
Testis volume	
Left (mL)	14.62 ± 3.78
Right (mL)	15.22 ± 2.62
LH (mU/mL)	7.46 ± 4.11
FSH (mU/mL)	19.36 ± 12.83
Prolactin (ng/mL)	8.07 ± 4.95
Total testosterone (ng/dL)	563.78 ± 87.66
Sperm volume (mL)	3.78 ± 1.08
Pathology diagnosis	
Normal spermatogenesis (%)	0 (0.0%)
Hypospermatogenesis (%)	38 (21.1%)
Maturation arrest, incomplete (%)	22 (12.2%)
SCOS, incomplete (%)	19 (10.5%)
Maturation arrest, complete (%)	24 (13.3%)
SCOS, complete (%)	68 (42.9%)

FSH: Follicle Stimulating Hormone; LH: Luteinizing Hormone; SCOS: Sertoli Cell Only Syndrome

**Table 2:** Comparing the baseline characteristics and laboratory values between those with successful and failed micro-TESE

	Successful Micro-TEST (n = 79)	Failed Micro-TESE (n = 92)	p-value
Age (years)	35.6 ± 8.4	33.6 ± 6.2	0.312
Testis volume			
Left (mL)	13.78 ± 2.34	15.19 ± 3.66	0.158
Right (mL)	15.93 ± 1.63	14.07 ± 5.49	0.122
LH (mU/mL)	7.86 ± 3.52	8.03 ± 2.34	0.205
FSH (mU/mL)	9.71 ± 7.32	29.03 ± 19.12	**<0.001**
Prolactin (ng/mL)	8.12 ± 1.47	8.51 ± 2.84	0.283
Total testosterone (ng/dL)	551.07 ± 96.4	588.23 ± 63.21	0.068
Sperm volume (mL)	4.01 ± 1.23	3.69 ± 1.88	0.981

**Figure 1:**
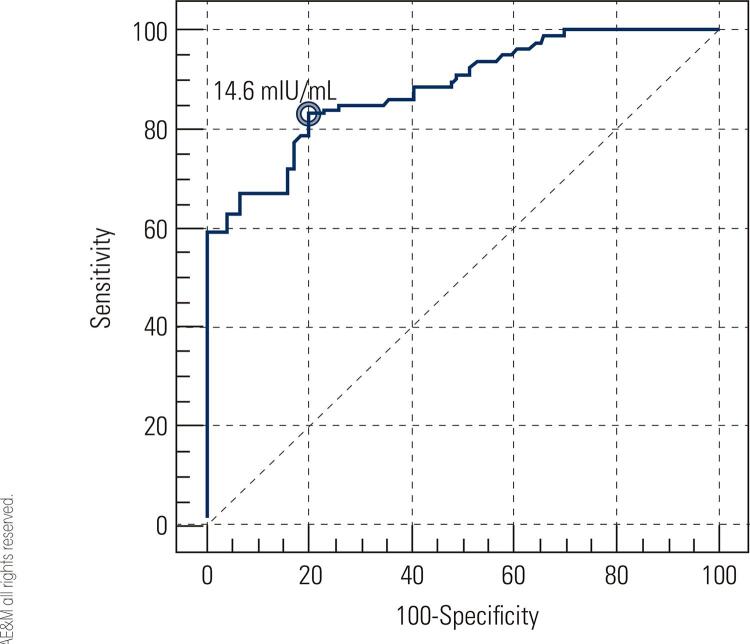
The ROC curve of follicle stimulating hormone (FSH), determining the cut-off value between successful or failed sperm retrieval.

FSH: Follicle Stimulating Hormone; LH: Luteinizing Hormone;

In order to determine the optimal cut-off points for FSH level, ROC curves were drawn. The ROC curve analysis for FSH level has been shown differentiate between these two groups (Figure 1). The area under the ROC curves was 0.88 [0.82-0.93], which is statistically significant (with a 95% CI). Accordingly, the cut-off value of FSH was 14.6 mIU/mL. At this value, the findings discriminated between groups A and B with sensitivity of 83.5% [73.5%-90.9%] and a specificity of 80.3% [69.5%-88.5%]. At this value, findings were PPV, 81.5%; NPV, 82.4; LR+, 4.23; and LR-, 0.21 (Table 3). When the cut-off values were increased to >14.6 mIU/mL, the sensitivity was increased and the specificity was decreased. Conversely, when the cut-off values were lesser than 14.6 mIU/mL, the sensitivity was decreased and the specificity was increased (Table 3).

**Table 3:** Serum FSH data and cut-off point, sensitivity, specificity and likelihood ratios (LR) for sperm retrieval

FSH (mU/mL) cut-off point, ≥	Sensitivity, %	Specificity, %	LR+	LR-
11	64.6	93.4	9.8	0.38
12	67.1	84.2	4.25	0.39
13	77.2	82.9	4.5	0.27
16	84.8	71.1	2.93	0.21
17	84.8	65.8	2.48	0.23
23	93.7	47.4	1.78	0.13
30	98.7	34.2	1.5	0.04

## DISCUSSION

In patients with NOA undergoing ICSI, micro-TESE is the method of choice for analyzing spermatozoa. Currently, the micro-TESE is considered the modality of choice for sperm retrieval in patients with NOA which is associated with minimal complications and adverse events because of microvascular preserving dissection (19,20). However, proposing non-invasive and more precise methods is essential for predicting successful spermatozoa retrieval especially in NOA patients. In the current study we tried to determine the predictive value of serum FSH level for micro-TESE in patients suffering from NOA. We found that a cut-off value of 14.6 mIU/mL for serum FSH level will predict the outcome of micro-TESE with a sensitivity and specificity of 83.5% [73.5%-90.9%] and 80.3% [69.5%-88.5%] respectively.

Several studies have investigated the predictive value of serum markers for NOA patients undergoing sperm retrieval. Tsujimura and cols. (21), demonstrated that serum levels of FSH, total T, and inhibin B were the most accurate predictors of successful sperm retrieval in patients with idiopathic NOA who underwent microdissection TESE. Christman and cols. (22) also investigated seven parameters, including semen volume, semen fructose, FSH, T, E2, PRL, and testicular atrophy in azoospermic patients and concluded that FSH was the best predictor of sperm retrieval in NOA patients. Similarly, Chen and cols. (9) revealed that among all the factors examined in their population, only FSH could determine the type of azoospermia. The area under the FSH ROC curve in the studies by Schoor and cols. (0.87) (23), Chen and cols. (0.85) (9), and Christman and cols. (0.84) (22) was favorably comparable to ours (0.88, with a 95% CI). These findings provide the predictive value of FSH for sperm retrieval techniques in patients with NOA. Tournaye and cols. (24) revealed that a two-fold rise above the upper border of normal serum FSH level was used as the cut-off value for spermatogenesis, but this value showed lower predictive value compared to histopathology. In addition, the successful sperm retrieval rate decreased from 77% to 29% in the azoospermic patients with small testicular size (< 4 cm) and elevated FSH levels (> 10 mIU/mL) (25). There is also data showing higher microTESE retrieval rates in smaller volume testes and data showing no difference in retrieval rates based on FSH (26-28). Some other data have also demonstrated higher retrieval rates in NOA patients with higher FSH levels (29).

Serum FSH values with an upper limit as high as 20 IU/mL have been indicated as the sperm retrieval success indicator in several studies (30). American Society for Reproductive Medicine also described more than twice the normal upper limit of serum FSH level as a dependable indicator of abnormal spermatogenesis (31). In the study by Christman and cols. (22), the cut-off point of FSH with the highest likelihood ratio was ≥ 12.3 mIU/mL. Chen and cols. (9) also concluded that increased plasma levels of FSH > 19.4 mIU/mL could be used as a predictive scale for sperm retrieval in ARTs. In our study, the cut-off point of FSH was 14.6 mIU/mL. Thus, the cut-off value for serum FSH is rather variable for predicting the success rate of sperm retrieval in azoospermic patients, and no agreement has been gained in this regard.

This inconsistency might be associated with the techniques used to obtain sperm. Microsurgical Testicular Sperm Retrieval (microTESE) has higher sperm retrieval rate compared to random testicular biopsy (9). Besides, lower chance of successful sperm retrieval has been reported by percutaneous fine needle aspiration compared to testicular sperm extraction by open biopsy in patients with NOA (20). Furthermore, application of bilateral testicular biopsy (32) and use of at least 6 number of biopsy sites have been recommended in order to retrieve spermatozoa in patients with NOA (32). Another major reason for the variability may be related to different causes of NOA. Schiff and cols. (33) showed that the mean FSH level of 33.2 IU/L represented a sperm retrieval rate of 72% per TESE attempt in patients with Klinefelter syndrome. Pening and cols. (34) also reported that among 143 men with NOA, 8% had Klinefelter syndrome and 9% had Y microdeletion, presenting 80% and 100% unsuccessful sperm recovery rates, respectively (34). Moreover, it has been described that in high FSH levels three folds above the normal limit (more than 27 mIU/mL), full spermatogenesis exists with a probability of 95%, but this cannot be used for diagnosis of Sertoli-cell-only syndrome and spermatogenic maturation arrest (35).

In the study by Carpi and cols. (36), sensitivity of FSH levels varied from 9% to 71% and their specificity from 40% to 90%. Besides, the findings of other studies demonstrated that increased FSH levels increased sensitivity and decreased specificity for predicting the sperm retrieval success rate (9). In a study by Chen and cols. (9), elevated FSH level (>19.4 mIU/mL) in azoospermic men was a predictive criterion for failure of sperm retrieval, with a probability of 100%, although the sensitivity of FSH level was 70%. Furthermore, when the cut-off point was 13.7 mIU/mL, the sensitivity and specificity of FSH level were 85.7% and 87.0%, respectively. Similarly, our results demonstrated that a cut-off point of FSH ≥ 14.6 mIU/mL led to the best sensitivity (83.5%) and specificity (80.3%) values.

We note some limitations to the current study. The main limitation of the present study was not differentiating the causes of NOA. Hence, more trials are needed to find responses to the existing controversies. The other limitation was that we did not measure the serum levels of inhibin B which is currently believed to be another sensitive predictor of the outcome of micro-TESE in patients with NOA. Further studies measuring these serum markers are currently underway in our center. The other limitation is that, a particular weakness of selecting a cut-off point as a predictor is that the cut-off point is not verified in a fresh group of men with NOA azoospermia. Finally, the number of included patients was low and calculating a cut-off value should be based on a larger series. However, we found that the power of the study was 80% to predict the outcome which is acceptable. Further studies with larger populations are recommended.

In conclusion, the results of the current study demonstrate that an FSH cut-off value of 14.6 mIU/mL predicts the outcome of micro-TESE in patients with NOA with high sensitivity (83.5%) and specificity (80.3%). This might be an effective and non-invasive, available and inexpensive method of predicting the outcome of sperm retrieval in patients with NOA.
